# *Nme8* is essential for protection against chemotherapy drug cisplatin-induced male reproductive toxicity in mice

**DOI:** 10.1038/s41419-024-07118-2

**Published:** 2024-10-06

**Authors:** Haixia Zhu, Hongxiang Wang, Dan Wang, Shuqiao Liu, Xiaoli Sun, Zhengjiang Qu, Aizhen Zhang, Chao Ye, Runze Li, Bin Wu, Min Liu, Jiangang Gao

**Affiliations:** 1 School of Life Science and Key Laboratory of the Ministry of Education for Experimental Teratology, Shandong University, Qingdao, China; 2 Department of Pharmacology, School of Pharmaceutical Sciences, Cheeloo College of Medicine, Shandong University, Jinan, China; 3 Obstetrics department, Qingdao Central Hospital, University of Health and Rehabilitation Sciences (Qingdao Central Hospital), Qingdao, China; 4 Medical Science and Technology Innovation Center, Shandong First Medical University, Jinan, China; 5 Department of Reproductive Medicine, Central Hospital Affiliated to Shandong First Medical University, Jinan, China; 6 Cheeloo College of Medicine, Shandong University, Jinan, China; 7 The Affiliated Taian City Central Hospital of Qingdao University, Taian, China

**Keywords:** Infertility, Autophagy

## Abstract

Cisplatin (CP), a chemotherapy drug commonly used in cancers treatment, causes serious reproductive toxicity. With younger cancer patients and increasing survival rates, it is important to preserve their reproductive capacity. NME8 is highly expressed in testis and contains thioredoxin and NDPK domains, suggesting it may be a target against the CP-induced reproductive toxicity. We deleted exons 6–7 of the *Nme8* in mice based on human mutation sites and observed impaired transcript splicing. In mice, *Nme8* was not essential for spermatogenesis, possibly due to functional compensation by its paralog, *Nme5*. *Nme8* expression was elevated and translocated to the nucleus in response to two weeks of CP treatment. Under CP treatment, *Nme8* deficiency further impaired antioxidant capacity, induced lipid peroxidation and increased ROS level, and failed to activate autophagy, resulting in aggravated DNA damage in testes and sperm. Consequently, the proliferation and differentiation of spermatogonia and the meiosis of spermatocyte were almost completely halted, and sperm motility was impaired. Our research indicates that NME8 protects against CP-induced testis and sperm damage. This may provide new insights into the physiological functions of the *Nme* family and potential targets for preserving fertility in young male cancer patients.

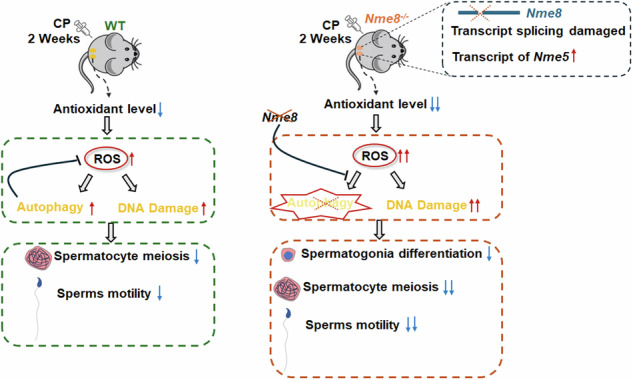

## Introduction

In the reproductive-age population, both the incidence and survival rates of cancer are rising [1, 2]. With improvements in early diagnosis and treatment options for cancer, an increasing number of young people undergoing these therapies are now becoming long-term cancer survivors [3, 4]. Males cannot regain fertility through hormone therapy after receiving high-dose chemotherapy [5]. Hence, Protecting the reproductive system of young cancer patients during treatment to mitigate the reproductive toxicity of chemotherapy and promote postoperative restoration of reproductive capability is of paramount importance [6].

Male fertility is maintained by ongoing spermatogenesis, an intricate process comprising three stages: proliferation and differentiation of spermatogonia, meiosis of spermatocytes, and the differentiation of round spermatids into mature spermatozoa [7]. Spermatogenic cells divide and differentiate rapidly, making them easily susceptible to chemotherapy drugs. Chemotherapy treatment in male cancer patients damages spermatogenic tubules [8, 9]. Platinum-based chemotherapeutic agents account for approximately 70% of all cancer treatment regimens, with cisplatin (CP) being the most commonly used to treat various cancers [10]. CP is effective in treating pediatric malignant tumors and improves the long-term survival rate of cancer patients, but it has been shown to cause severe testicular damage [11, 12]. After CP exposure, oxidative stress and membrane lipid peroxidation occur in the testes due to an imbalance in antioxidant status and the accumulation of reactive oxygen species (ROS). This cause damage to the cell membrane, leading to reproductive toxicity [13]. Therefore, protecting the testis from ROS accumulation and DNA damage during chemotherapy is crucial for maintaining fertility in chemotherapy patients.

*Nme8* (non-metastatic family member 8) was categorized as a member of the Nme family and previously identified as sptrx-2 and txndc3 [14]. In humans, *NME8* has been reported as a gene implicated in primary ciliary dyskinesia, a genetic condition characterized by chronic respiratory tract infections, left-right asymmetry randomization, and male infertility [15]. The NME8 protein comprises three C-terminal domains of nucleoside diphosphate kinase (NDPK) and an N-terminal thioredoxin domain. Thioredoxin is an antioxidant that maintain intracellular redox stability by reducing protein disulfides and is also involved in DNA and protein repair by regulating redox-dependent signal pathways [16]. NME8 additionally displays a 3′-5′ exonuclease activity [17], suggesting it participants in DNA repair and proofreading. The NME8 protein is specifically expressed in the testis of mice and binds to the fibrous sheath of the sperm during the final stages of sperm tail maturation [18]. Based on the expression pattern and protein structure of the NME8, this study aims to determine whether NME8 plays a role in spermatogenesis and reproductive toxicity induced by CP.

In this study, we found NME8 responded to CP stimulation in the testis of mice. We then generated *Nme8* knockout (*Nme8*^*−/−*^) mice and tested the role of NME8 in antioxidation and DNA repair in the CP-treatment mice. This study not only enriches the understanding of NME8 function but also provides a potential new approach for maintenance reproductive ability in young male cancer patients after chemotherapy.

## Results

### NME8 responded to CP stimulation in the testis

**Fig. 1:**
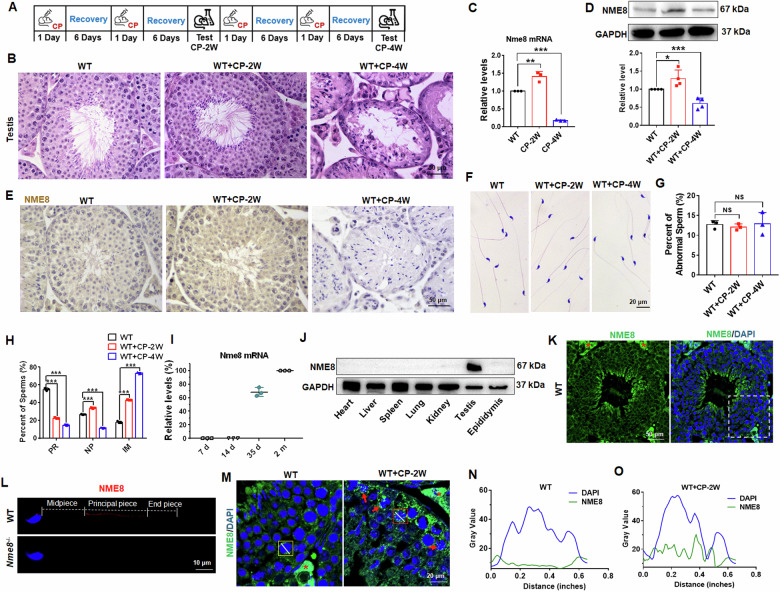
The NME8 expression in response to CP stimulation. **A** Schematic diagram of CP administration in mice. **B** The H&E staining of mice testis at different CP stimulation time points. Scale bar = 50 μm. **C** Changes in *Nme8* mRNA expression in the mouse testis at different administration times. *n* = 3 biologically independent mice. **D** The protein expression of NME8 in the testis of mice. *n* = 4 biologically independent mice. **E** Immunohistochemistry result of Nme8 protein in seminiferous tubules. Scale bar = 50 μm. **F** The H&E staining of sperm from cauda epididymis of mice in different groups. **G** Statistical result for the percent of abnormal sperms according to H&E staining. *n* = 3 biologically independent mice. **H** CASA analysis was used to detect sperm motility. PR, progressive motility; NP, nonprogressive motility; IM, immobilized sperm. *n* = 3 biologically independent mice. **I** The mRNA level of *Nme8* was examined in testis of mice at different times after birth. *n* = 3 biologically independent mice. **J** Expression of the NME8 protein in different tissues of adult mice. **K**, **L** Immunofluorescence was used to test the location of NME8 in mice testis and sperms. **K** Scale bar = 50 μm. Asterisks indicate non-specific antibody binding. The negative control was shown in Fig. S1B. **L** Scale bar = 10 μm. **M** Changes in the localization of NME8 after two weeks of CP stimulation. The left panel shows an enlarged view of the white dashed box in (**K**); the red arrows point to cells in which NME8 translocated to the nucleus. Scale bar = 20 μm. **N**, **O** Fluorescence co-localization in the nucleus was analyzed using Image J, along the direction of the solid white line in the yellow and red boxes in (**M**). *P < 0 .05, **P < 0.01, ***P < 0.001, NS indicates non-significant.

To investigate the role of NME8 under CP exposure, we established pharmacological models based on existing literature [19]. Wild-type (WT) mice were divided into three groups: one group received intraperitoneal injections of normal saline for 4 weeks, while the CP groups received injections for 2 and 4 weeks, respectively (Fig. 1A). In the WT group, germ cells were closely and regularly arranged in the seminiferous tubules, with no structural abnormalities observed in the CP-2W group (Fig. 1B). However, the CP-4W group showed notable germ cell loss and damage (Fig. 1B). We tested Nme8 expression and found that both mRNA and protein levels were upregulated after twice CP treatments followed by six days of recovery for each but downregulated after the same CP treatment and recovery four times (Fig. 1C, D). Immunohistochemical staining for NME8 confirmed these results (Fig. 1E), suggesting that NME8 may plays a role in the CP-2W group testis. The almost complete loss of germ cells in the 4-week CP treatment group prevented us from studying the function of NME8, so the 2-week CP treatment was selected as the pharmacological model for subsequent experiments. Neither 2 nor 4 weeks of CP treatment affected sperm morphology in the cauda epididymis (Fig. 1F, G), but both treatments reduced sperm motility (Fig. 1H).

### NME8 translocated into the nucleus following CP stimulation

We examined the expression pattern of *Nme8* in WT mice testes, and the mRNA was barely detectable at 7 and 14 days of age (Fig. 1I). In adult mice, NME8 protein was detected abundantly in testicular tissue (Fig. 1J). Consistent with previous studies [5], NME8 was primarily expressed in deformed sperm, though other types of germ cells were not excluded (Fig. 1K). In sperms, NME8 was detected in the principal piece (Fig. 1L). And when the protein sample size of epididymis was elevated, a faint NME8 band could be observed in epididymis (Fig. S1A). In spermatocytes of WT mice, NME8 was expressed in the cytoplasm (Fig. 1M). However, after CP treatment, NME8 protein co-localized with DAPI in some spermatocytes (Fig. 1M). We used Image J software to analyze the fluorescence at the position of the white line segment in Fig. 1E. The results showed that there was no co-expression of DAPI and NME8 in spermatocytes of WT mice, while there was a clear overlap of DAPI and NME8 fluorescence in spermatocytes of the CP group mice (Fig. 1N, O). These results suggested that NME8 may enter the nucleus under CP treatment.

### Deletion of *Nme8* has no effect on the reproductive ability of male mice

**Fig. 2:**
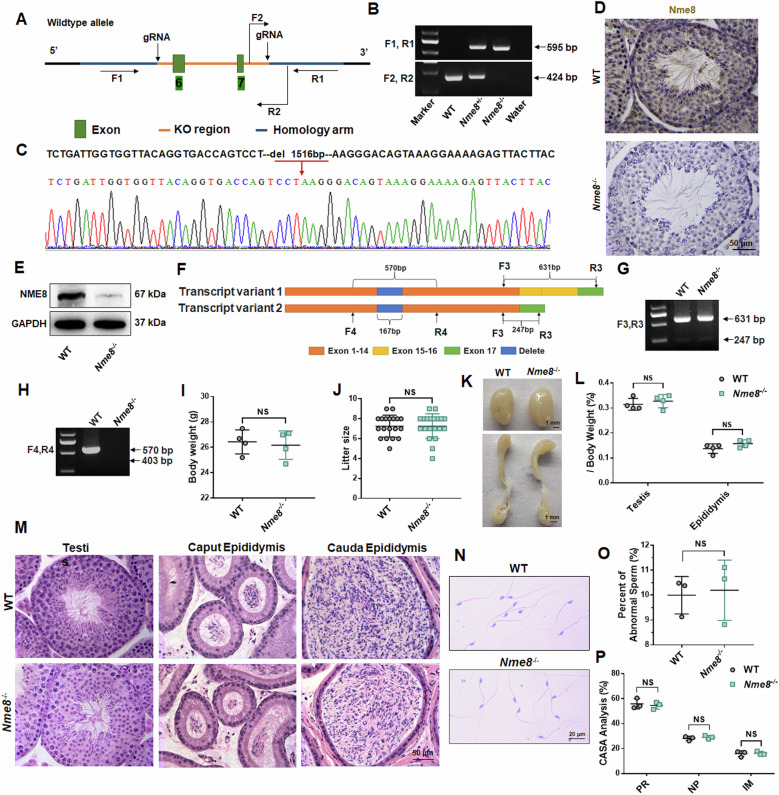
*Nme8*^*−/−*^ male mice exhibited normal fertility. **A** Construction diagram of *Nme8*^*−/−*^ mice. **B** PCR was performed to determine mice genotypes. **C** The sequencing results of the PCR product from *Nme8*^*−/−*^mice. **D**, **E** Immunohistochemistry analysis and western blot confirmed successful knockout of the NME8 protein. Scale bar = 50 μm. **F** Schematic illustration of two transcripts of *Nme8*. **G**, **H** Agarose gel electrophoresis results of the PCR products. **I** Body weights of adult WT and *Nme8*^*−/−*^ mice. *n* = 4 biologically independent mice. **J** The number of offspring from WT or *Nme8*^*−/−*^ male mice by cohabiting with WT female mice. *n* = 3 biologically independent mice. **K**, **L** The morphology and weights of the testis and epididymis were assessed in adult WT and *Nme8*^*−/−*^ mice. Scale bar = 1 mm. **I**, **J**, and **L** NS indicates non-significant. **M**, **N** H&E staining was used to examine the testis, epididymis, and sperms of adult mice. Scale bar = 50 μm. **O**, **P** Sperm deformity and motility were evaluated in WT and *Nme8*^*−/−*^ mice. NS indicates non-significant, *n* = 3 biologically independent mice.

To investigate the role of *Nme8* in the testis, we employed CRISPR/Cas9 technology to generate *Nme8*^*−/−*^ mice. A mutation in intron 6 between exons 6 and 7 in the human *NME8* gene affects the ratio of two physiological *Nme8* transcripts [15]. Thus, we designed two pairs of gRNAs to delete exons 6-7 of the *Nme8* gene (Fig. 2A) and utilized PCR to identify deletion mutations (Fig. 2B). DNA sequencing revealed a 1516 bp deletion in the *Nme8*^*−/−*^ mice genome sequence (Fig. 2C). Immunohistochemistry staining and western blot results confirmed the successful knockout of the NME8 protein in the testis (Fig. 2D, E), with no expression of NME8 in the sperm of *Nme8*^*−/−*^ mice (Fig. 1L). These findings indicated the successful generation of *Nme8*^*−/−*^ mice. To examine whether *Nme8* transcript splicing is defective and to determine the ratio of the two transcripts, we amplified the *Nme8* cDNA fragments spanning different positions of transcripts 1 and 2 using F3 and R3, and the fragments spanning the deleted region using primers F4 and R4 (Fig. 2F). Agarose gel results showed no difference in bands and brightness between WT and *Nme8*^*−/−*^ mice when F3 and R3 were used as primers, indicating no change in the ratio of the two transcripts in *Nme8*^*−/−*^ mice (Fig. 2G). When F4 and R4 were used as primers, WT mice showed a 570 bp PCR product, while *Nme8*^*−/−*^ mice did not show any band, indicating that the knockout of exons 6-7 affected the splicing of the *Nme8* transcript (Fig. 2H). Then, we designed primers (F5, R5 in Table S2) to sequence the transcript following the deletion site and used Swiss model (https://swissmodel.expasy.org/) to predict the protein structure. It revealed the absence of NDPK domains of NME8 in *Nme8*^*−/−*^ mice (Fig. S2A).

We found that the reproductive capacity of heterozygous mutant mice was unchanged, and the offspring genotypes conformed to Mendelian inheritance. Compared with WT mice, the daily behavior and body weight of *Nme8*^*−/−*^ adult male mice showed no difference (Fig. 2I). We assessed the reproductive capacity of male mice by caging WT and *Nme8*^*−/−*^ adult males with WT females for three months and counting the litters. No difference in litter size was observed between *Nme8*^*−/−*^ and WT mice (Fig. 2J). The morphology and the ratio of testes and epididymis weight to body weight remained unchanged after *Nme8* knockout (Fig. 2K, L). In *Nme8*^*−/−*^ adult male mice, H&E staining showed normal testis structure and epididymal epithelium (Fig. 2M), and sperm morphology and motility in the cauda epididymis were also normal (Fig. 2N–P). These results indicated that the *Nme8* knockout does not affect the reproductive ability of male mice. To explore whether other Nme family members play a compensatory role after Nme8 knockout, we tested *Nme5* and *Nme6*, which contain NDPK domains and are highly expressed in the testis, as well as *Nme9*, which is predicted to substitute functionally for *Nme8* [15, 18]. We found that the mRNA level of *Nme5* was highest in mouse testis, followed by *Nme8* and *Nme6*, with very low levels of *Nme9* (Fig. S2B). Moreover, only the mRNA level of *Nme5* increased (Fig. S2C). This suggested that *Nme5* may play a compensatory role in the absence of *Nme8*.

### *Nme8* knockout led to testis injury under CP treatment

**Fig. 3:**
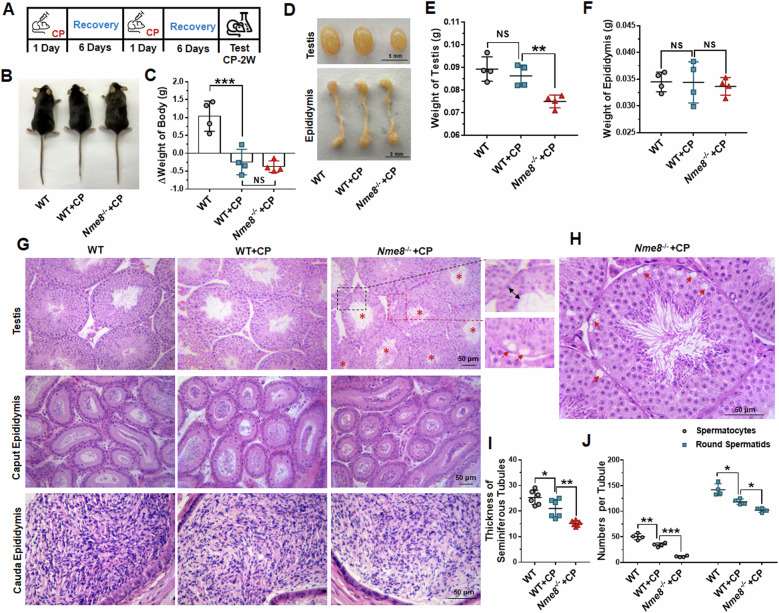
Deletion of *Nme8* leads to testis damage after CP stimulation for 2 weeks. **A** Schematic of the administration method and timeline. **B** Body image of mice after CP injection for 2 weeks. **C** Changes in body weight of mice before and after CP administration. *n* = 4 biologically independent mice. **D**–**F** The morphology and weights of the testis and epididymis in different groups. Scale bar = 5 mm. *n* = 4 biologically independent mice. **G** H&E staining was performed for the testis and epididymis of different groups of mice. The enlarged image inside the black and red dotted boxes is on the right; the red asterisk marked the seminiferous tubule in which no spermatocyte was observed; black double arrows indicated the thickness of the seminiferous tubule; red arrows indicated vacuoles. Scale bar = 50 μm. **H** Representative diagram of vacuoles in the seminiferous tubules of *Nme8*^*−/−*^+CP mice. Scale bar = 50 μm. **I** Statistical results of the thickness of the seminiferous tubules. *n* = 6 biologically independent mice. **J** Statistics on the number of spermatocytes and round spermatids in per tubule. *n* = 4 biologically independent mice. *P < 0 .05, **P < 0.01, ***P < 0.001, NS indicates non-significant.

To explore the role of NME8 in response to CP stimulation, WT and *Nme8*^*−/−*^ mice were treated with CP for two weeks at the established dosage (Fig. 3A). At the end of the two-week period, the body weight of mice in the WT + CP group decreased compared with WT mice, with no differences observed between the WT + CP and *Nme8*^*−/−*^+CP groups (Fig. 3B, C). After CP treatment, the size and weight of testes from WT mice did not change, while there was a significant decrease in *Nme8*^*−/−*^ mice (Fig. 3D, E). CP stimulation had no effect on the size and weight of the epididymis in WT and *Nme8*^*−/−*^ mice (Fig. 3D, F). H&E staining of paraffin sections of the testis and epididymis showed that *Nme8*^*−/−*^+CP mice exhibited a sparse arrangement of germ cells compared to the other groups (Fig. 3G). CP administration decreased the seminiferous tubule thickness of WT mice (Fig. 3I). Compared with WT + CP mice, the thickness of seminiferous tubules in *Nme8*^*−/−*^+CP mice decreased (Fig. 3G, I). In *Nme8*^*−/−*^+CP mice, we observed vacuoles and the absence of spermatocytes at the edges of most seminiferous tubules, suggesting a phenomenon of cell loss (Fig. 3G, H). Based on the H&E staining results, we counted spermatocytes and round spermatids in individual seminiferous tubules. The results showed that the number of spermatocytes and round spermatids decreased in WT mice after CP stimulation and further decreased in *Nme8*^*−/−*^+CP mice (Fig. 3J).

### Deletion of *Nme8* significantly decreased sperm motility when stimulated with CP

**Fig. 4:**
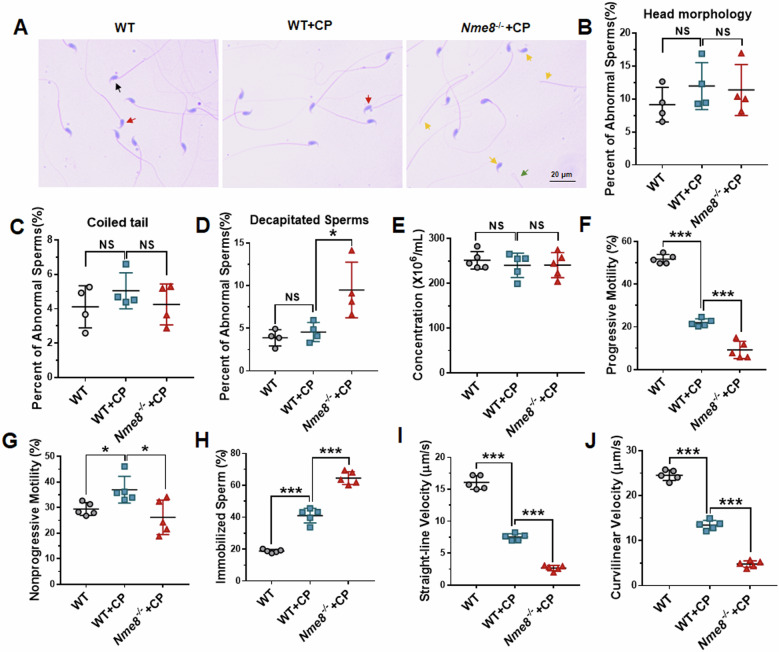
The impact of *Nme8* deletion on sperms under CP stimulation was tested. **A** H&E staining was performed on a smear of sperms from the epididymis cauda. Scale bar = 20 μm. The black arrows point to normal sperm, and the red, green, and yellow arrows point to abnormal head, coiled tail, and decapitated sperm, respectively. **B**–**D** The percent of abnormal sperms was calculated. **E** The concentration of sperms in different groups of mice under the same treatment condition. **F**–**H** The CASA analysis was used to test sperm motility, including progressive motility, nonprogressive motility, and immobilized sperms. **I**, **J** The straight-line and curvilinear velocity of sperms were tested. *P < 0 .05, ***P < 0.001, NS indicates non-significant, *n* = 4 biologically independent mice in each group.

NME8 integrates into the sperm tail during the final stage of spermatogenesis. To understand its role in sperm, we examined sperm morphology in the cauda epididymis of mice using H&E staining (Fig. 4A). We found that CP stimulation did not affect the sperms morphology in WT mice, but significantly increased the proportion of decapitated sperms after two weeks of CP treatment in *Nme8*^*−/−*^ mice (Fig. 4B–D). Additionally, CASA analysis showed no difference in sperm concentration among WT, WT + CP, and *Nme8*^*−/−*^+CP mice (Fig. 4E). However, after CP treatment, WT mice exhibited reduced progressive motility and increased nonprogressive motility and immobilized sperms (Fig. 4F–H). Notably, *Nme8*^*−/−*^+CP mice showed further reductions in progressive motility and increased immobilized sperms compared to WT + CP mice (Fig. 4F–H). Moreover, CP stimulation reduced both straight-line and curvilinear velocity of sperms in WT mice, with further decreases observed after *Nme8* knockout (Fig. 4I, J). These results indicated that while CP primarily affects sperm motility in WT mice, *Nme8* deletion exacerbates these effects.

### Spermatogonia and spermatocytes decreased in *Nme8*^*−/−*^+CP mice testis

**Fig. 5:**
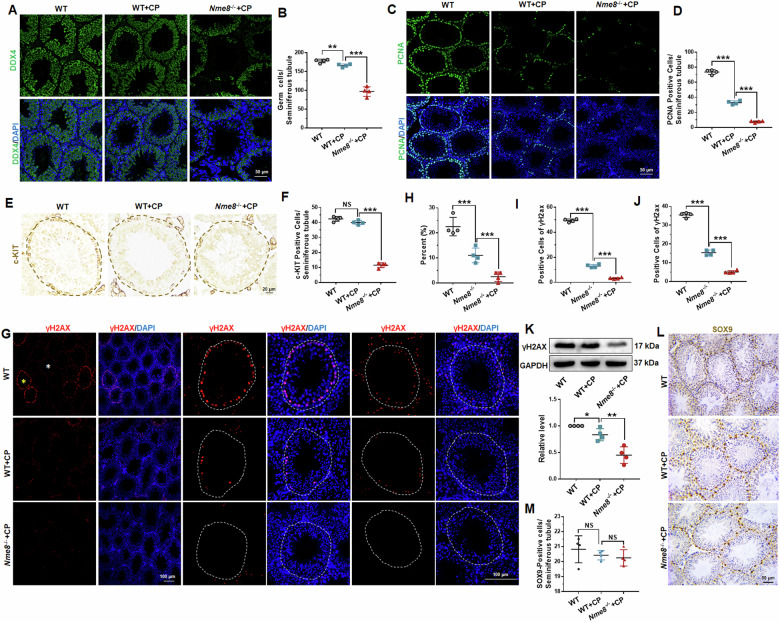
*Nme8* deficiency affects spermatogonia differentiation and spermatocyte meiosis under CP stimulation. **A**, **B** The immunofluorescence staining and statistical results of DDX4. Scale bar = 50 μm. **C**, **D** The immunofluorescence staining and statistical results for PCNA in mice testis. Scale bar = 50 μm. **E**, **F** Immunohistochemistry of c-KIT was used to test differentiating spermatogonia. Scale bar = 20 μm. **G** γH2AX was used to test the spermatocyte meiosis in the testis. Scale bar = 100 μm. The yellow and white asterisks marked the tubules that abundantly and punctately express γH2AX, respectively. **H** The proportion of tubes with extensive γH2AX expression in all tubes. **I**, **J** The number of cells with extensive (**I**) and punctate γH2AX (**J**) in each tubule. **K** The protein expression of γH2AX in the testis of different groups of mice. **L**, **M** SOX9 was used to mark Sertoli cells in testis. *P < 0 .05, **P < 0.01, ***P < 0.001, NS indicates non-significant, *n* = 4 biologically independent mice in each group.

To further determine the types of lost cells, DDX4 was used to label germ cells in the seminiferous tubules. The results showed that WT mice had reduced germ cell numbers after two weeks of CP treatment (Fig. 5A, B). *Nme8*^*−/−*^ mice exhibited a significant decrease in germ cells after CP treatment compared to WT mice (Fig. 5A, B). Next, we labeled proliferating spermatogonia and spermatocytes in the testis with PCNA [20]. The number of PCNA-positive cells in individual seminiferous tubules was reduced in the WT + CP group and significantly lower in the *Nme8*^*−/−*^+CP group (Fig. 5C, D). c-KIT was used to test the differentiation of spermatogonia, and the number of c-KIT positive cells in the seminiferous tubules with active spermatogonia differentiation was counted as described in our previous article [20]. We found that CP had no effect on the number of c-KIT positive cells in WT mice but reduced the number of differentiation spermatogonia in the seminiferous tubules of *Nme8*^*−/−*^ mice (Fig. 5E, F). γH2AX, a double-strand break (DSB) repair protein [21], was also used to detect spermatocytes in the testis [22]. At the initiation of meiosis, it is expressed abundantly at the edge of the seminiferous tubules (yellow asterisk in Fig. 5G); and at the pachytene stage, these double-strand breaks are resolved by homologous recombination, leaving γH2AX in a punctate form only on the XY body (white asterisk in Fig. 5G). The statistical results showed that the proportion of seminiferous tubules extensively stained with γH2AX decreased in WT + CP mice and further significantly decreased in *Nme8*^*−/−*^+CP mice (Fig. 5H). The number of spermatocytes with extensive and punctate γH2AX expression in individual seminiferous tubules showed similar results: decreasing after CP stimulation and further decreasing with *NME8* knockout (Fig. 5I, J). The protein level of γH2AX in testis decreased in the WT + CP group and further significantly decreased in *Nme8*^*−/−*^+CP group (Fig. 5K). In addition to germ cells, Sertoli cells also provide a favorable environment for spermatogenesis [22]. We labeled Sertoli cells in the testis with immunohistochemical staining of SOX9 and found that the number of Sertoli cells did not change after CP treatment in either WT or *Nme8*^*−/−*^ mice (Fig. 5L, M). These results indicated that *Nme8* deficiency caused a decrease in the numbers of spermatogonia and spermatocytes under CP treatment.

### Redox imbalance in the testis due to *Nme8* deficiency

**Fig. 6:**
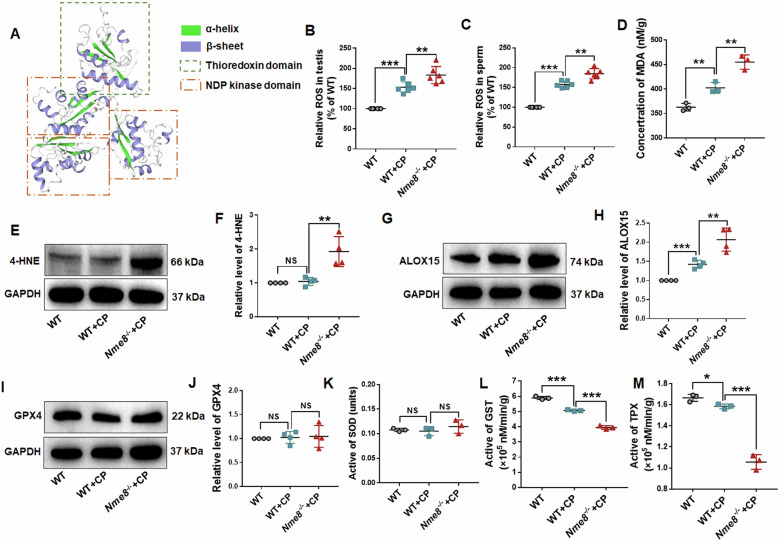
An imbalance in the redox system was observed in the testes of *Nme8*^*−/−*^ mice stimulated with CP. **A** Protein structure diagram of NME8. ROS activity was examined in the (**B**) testis and (**C**) sperms. **D** The concentration of MDA in the testis was measured. **E**, **F** Western blot results for 4-hydroxynonenal (4-HNE) were obtained from the testes of WT, WT + CP, and *Nme8*^*−/−*^+CP mice. **G**, **H** The proteins ALOX15 and (**I**, **J**) GPX4 were tested in the testis by western blot. The activity of SOD, GST, and TPX were measured by Elisa assay in the testes of WT, WT + CP, and *Nme8*^*−/−*^+CP mice, respectively. *P < 0 .05, **P < 0.01, ***P < 0.001, NS indicates non-significant, *n* = 4 biologically independent mice in each group.

**Fig. 7:**
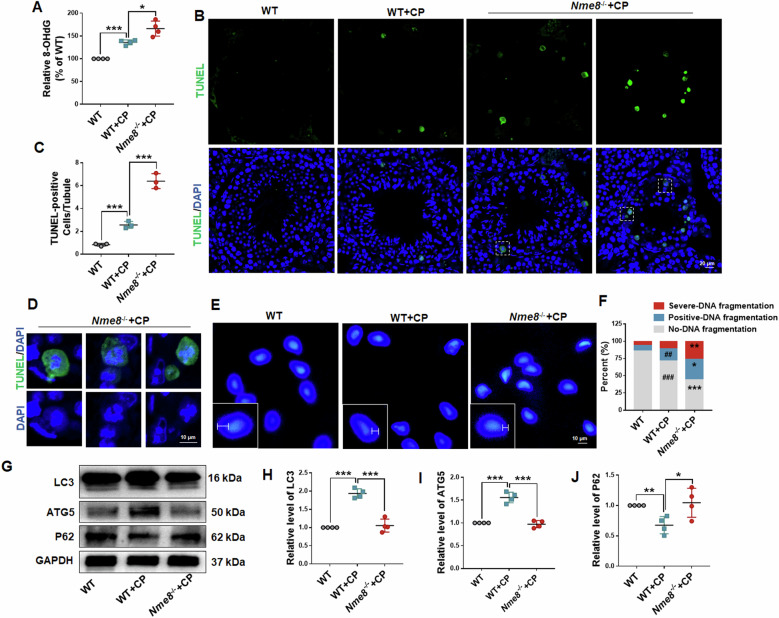
The deletion of *Nme8* results in elevated DNA damage in the testes and sperm after CP stimulation. **A** The relative levels of 8-OHdG in the testes of WT, WT + CP, and *Nme8*^*−/−*^+CP mice were measured. *n* = 3 biologically independent mice in each group. **B**, **C** The TUNEL assay was used to test the DNA damage in the testis. Scale bar = 20 μm, *n* = 4 biologically independent mice in each group. **D** The enlarged view inside the white dotted box in (**B**). Scale bar = 20 μm. **E**, **F** SCD was used to analyze DNA damage in sperms from WT, WT + CP, and *Nme8*^*−/−*^+CP mice, and the degree of DNA fragmentation was assessed by comparing halo and nuclear sizes. Scale bar = 10 μm. ^##^*P* < 0.01, ^###^*P* < 0.001 *vs* WT; *P < 0 .05, **P < 0.01, ***P < 0.001 *vs* WT + CP; *n* = 4 biologically independent mice in each group. **G**–**J** The autophagy related proteins LC3, ATG5, and P62 were tested in the testis. *P < 0 .05, **P < 0.01, ***P < 0.001, *n* = 4 biologically independent mice in each group.

CP treatment increases levels of free radicals, especially ROS [23]. NME8, comprising an N-terminal thioredoxin domain and a C-terminal domain with three NDPK domain repeats [14, 18], as shown in the structure diagram predicted by the website (https://www.uniprot.org) [24] (Fig. 6A). Thioredoxin assists in rapid ROS removal by providing electrons to peroxiredoxins [25, 26]. Therefore, we examined ROS levels in the testes and sperms of WT and *Nme8*^*−/−*^ mice following two weeks of CP treatment. ROS levels were significantly elevated in both the testes and sperms of WT mice and exacerbated in *Nme8*^*−/−*^ mice (Fig. 6B, C). Elevated ROS disrupts intracellular redox balance, leading to lipid peroxidation and subsequent membrane damage [27]. The products of intracellular lipid peroxidation, malonaldehyde (MDA) and 4-hydroxynonenal (4-HNE) were tested. We observed elevated MDA levels in WT + CP mice and further increases in *Nme8*^*−/−*^+CP mice (Fig. 6D). The protein level of 4-HNE in the testis of WT + CP mice did not change but was elevated in *Nme8*^*−/−*^+CP mice testis (Fig. 6E, F). Arachidonate 15-lipoxygenase (ALOX15), one of the key enzymes involved in the production of 4-HNE [28], was upregulated in the testis of WT + CP mice approximately 1.4 times compared to WT mice, but increased to about 2.1 times in *Nme8*^*−/−*^+CP mice (Fig. 6G, H). The balance between ROS and antioxidant enzymes essential for producing healthy sperm and maintaining sperm quality [29]. Despite these changes, levels of antioxidant enzymes glutathione peroxidase 4 (GPX4) and superoxide dismutase (SOD) remained consistent across all groups (Fig. 7I, K). However, the actives of glutathione S-transferases (GST) and Thioredoxin peroxidase (TPx) decreased in the testis of WT + CP mice, and further reduced in *Nme8*^*−/−*^+CP mice (Fig. 7L, M). These results underscore the role of *Nme8* in maintaining redox homeostasis under oxidative stress.

### Deficient autophagy activation in *Nme8*^*−/−*^ mice caused further DNA damage

ROS is a major source of DNA damage that prompts NME8 to translocate to the nucleus in response to CP, considering the 3 ′-5′ exonuclease activity of NME8. So we checked for DNA damage in the testes and sperm. The levels of 8-hydroxy-2 deoxyguanosine (8-OHdG), a marker of oxidative DNA damage [30], were measured. The result showed that the 8-OHdG level increased following CP stimulation in the testis of WT mice and was further elevated after *Nme8* deletion (Fig. 7A). The TUNEL assay was used to test DNA damage using terminal deoxynucleotidyl transferase to catalyze the attachment of modified deoxynucleotides to DNA strand breaks [30]. In the testes of WT + CP mice, TUNEL-positive cells in individual seminiferous tubules were increased compared with WT mice (Fig. 7B, C). Moreover, *Nme8*^*−/−*^+CP mice showed a significant increase in TUNEL-positive cells compared with WT + CP mice (Fig. 7B, C). We found that the apoptotic signals appeared mainly at the edge of the seminiferous tubules, and most of them were dividing spermatocytes based on cell morphology (Fig. 7B, D). SCD assay was used to analyze DNA fragmentation in sperm [31]. The DNA fragmentation of sperm was categorized into three grades based on the width of the halo, and the percentage of sperms in each grade was counted separately. In WT mice, the proportion of sperms with positive-DNA fragmentation increased after CP stimulation (Fig. 7E, F). Compared with WT, *Nme8*^*−/−*^ mice showed a further increase in the proportion of sperms with positive-DNA fragmentation after CP stimulation, and a significant increase in severe-DNA fragmented sperms (Fig. 7E, F). *Nme8* deficiency led to aggravated DNA damage in the testis and sperm.

ROS are known to activate autophagy, which can inhibit DNA damage by maintaining the energy requirements necessary to support DNA repair processes [32, 33]. In our study, *Nme8* deficiency leads to aggravated DNA damage in the testes and sperms of mice. To explore whether autophagy was responsible for the increased DNA damage in *Nme8*^*−/−*^+CP mice, we examined autophagy-related proteins in the testis. After CP stimulation for two weeks, the expression of LC3 and ATG5 increased, P62 expression decreased, suggesting that autophagy was activated in WT + CP mouse testes (Fig. 7G–J). However, compared with WT + CP mice, the protein levels of LC3 and ATG5 significantly decreased, while the protein level of P62 increased in the testis of *Nme8*^*−/−*^+CP mice, all of which were essentially the same as those in WT mice (Fig. 7G–J). These findings suggest that *Nme8* deficiency impairs autophagy activation, exacerbating DNA damage under CP-induced oxidative stress.

## Discussion

As cancer mortality declines and the incidence of cancer in young people increases [34, 35], minimizing reproductive toxicity during treatment becomes increasingly important. The efficacy and safety of antioxidants used by patients in clinical trials has not been satisfactory [36], indicating a need to identify in vivo targets resistant to chemotherapy-induced damage. In this study, we used CRISPR/Cas9 technology to construct knockout mice with testis-specific expression of the *Nme8* gene. We found that while the reproductive ability of adult *Nme8*^*−/−*^ male mice was not affected, testis damage was aggravated by CP stimulation compared to normal mice. These findings suggest that *Nme8* is a conditionally essential gene during spermatogenesis in mice. Conditionally essential genes play crucial roles in biological adaptation to environmental changes and stress response. They are increasingly valued in targeted therapies under complex conditions, such as cancer therapy [37].

The structure of NME8 is similar in humans and mice, both containing one thioredoxin and three NDP-kinase domains, though differences in localization and function cannot be ruled out. In humans, NME8 is highly expressed in the testis and other tissues such as respiratory epithelial cells, localized from the caudal region of the head to the end of the principal piece of the sperm tail [14]. Two mutations in the *NME8* gene (a nonsense mutation (p.Leu426X) and a common intronic variant (c.271–27 C > T)) affect the ratio of two physiological *NME8* transcripts by alternative splicing, resulting in primary ciliary dyskinesia (PCD) [15]. In mice, NME8 is specifically expressed in the testis, localized in the principal piece of the sperm tail, and incorporated into the fibrous sheaths [18, 38]. An amino acid residue in exon 4 of NME8 was mutated to inactivate the thioredoxin domain, which did not affect mouse fertility [39]. In this study, we deleted exons 6-7 of *Nme8*, affecting the splicing of the *Nme8* transcript. Fertility and sperm quality were not affected in our *Nme8*^*−/−*^ male mice, suggesting that although NME8 is specifically expressed in the testis, it is not essential for spermatogenesis, possibly due to *Nme5* playing a substitutive role. To further confirm the compensatory role of *Nme5*, we will construct and inject the *Nme5* knockdown adenovirus into *Nme8*^*−/−*^ mice and then test whether *Nme8*^*−/−*^ mice exhibit testis damage. In addition, *Nme8*^*−/−*^+CP mice will be injected with adenovirus overexpressing *Nme5* to test whether sperm quality is improved. Furthermore, mutations in *Nme5* cause primary ciliary dysmotility in humans [40], and knockout mice exhibit skull depression, hydrocephalus and sperm flagella defects [41], suggesting *Nme5*’s essential role in cilia formation and function, including sperm flagella. Unlike NME8, NME5 only contains NDPK domains [42]. This indicated that the NDPK domain plays an important role in spermatogenesis and sperm flagellar function in male mice.

In the CP-induced testis injury model, NME8 expression level in the testes was elevated after two weeks of weekly CP treatment, suggesting its role in response to reproductive toxicity. Compared with WT + CP mice, the more severe arrest of spermatogonia proliferation and spermatocyte meiosis were shown in *Nme8*^*−/−*^+CP mice, leading to vacuolization in the seminiferous tubules. And *Nme8* knockout resulted in a greater decrease in sperm motility induced by CP, including reduced progressive motility, straight-line and curvilinear velocity. These results suggest that the loss of *Nme8* exacerbates the reproductive toxicity induced by CP. And the absence of *Nme8* significantly reduced γH2AX protein level, suggesting it may play a role in γH2AX protein stability. Furthermore, CP treatment did not affect Sertoli cell numbers. But the functional damage of Sertoli cells cannot be ruled out in CP treatment mice, such as disruption of the Sertoli cell tight junctions [43] and decreased production of transferrin, androgen-binding protein, lactate, and estradiol in Sertoli cells [44]. Since the NDPK domain is involved in spermatogenesis and regulates oxidative stress signaling pathways [45–47], NME5 containing the NDPK domain may play an antioxidant role in *Nme8*^*−/−*^+CP mice. While the increased *Nme5* could not recover the testis damage induced by CP, maybe because NME5 has no thioredoxin domain and the compensatory ability is limited, which may not be sufficient to resist CP-induced testis injury.

We analyzed the specific mechanisms based on the protein structure of NME8 under CP treatment. In WT mice, two weeks of CP stimulation led to decreased GSH and TPX activities and elevated ROS levels in mouse testes and sperm. It has been demonstrated that autophagy is induced by oxidative stress to simultaneously reduce ROS concentrations (upstream cause) and mitigate oxidative damage to biomolecules and organelles (downstream effect) [33]. The activation of autophagy in the WT + CP testis may inhibit ROS accumulation and attenuate DNA damage, thereby delaying testis damage in mice. Upon CP stimulation, *Nme8* knockout mice showed further decreased antioxidant capacity, increased lipid peroxidation, resulting in significantly higher MDA and ROS level in testis. This may be attributed to that autophagy is not activated by ROS in the testes of *Nme8*^*−/−*^+CP mice, exacerbating ROS accumulation and DNA damage, and leading to aggravated reproductive toxicity.

Our study also provides insights into understanding the physiological functions of Nme family proteins. In the Nme family, it has been proven that NME1, NME2 and NME4 can translocate to the cell nucleus [48, 49]. Their nuclear entry appears to be a response to environmental stimuli, followed by non-specific DNA binding [50]. *Nme1* and *Nme3* are implicated in the repair of DNA single-strand and double-strand breaks, and after removing the mitochondrial localization sequence [51]. We observed NME8 translocation to the nucleus in response to CP stimulation, accompanied by severe DNA damage in spermatocytes and sperms of *Nme8*^*−/−*^+CP mice, suggesting that NME8 play a role in DNA damage repair. Besides, NME4 can specifically eliminate defective mitochondria or cells through mitochondrial autophagy [52]. In the testis of *Nme8*^*−/−*^+CP mice, autophagy was not activated, indicating that NME8 may be crucial for autophagy activation. Furthermore, our results indicated decreased antioxidant capacity in the testes of *Nme8*^*−/−*^+CP mice. Although, whether NME8 exerts a direct antioxidant function via its thioredoxin domain remains to be investigated, its role in maintaining antioxidant capacity is noteworthy.

In this study, we analyzed the reason that *Nme8* knockout does not affect male fertility in mice, and explored the specific mechanisms that *Nme8* in response to chemotherapeutic agents CP. These not only adds insight into the function of the Nme family but may also provide potential new targets for maintaining fertility in young male cancer patients. In the future, we will continue to construct NME8 overexpression plasmid to further verify the protective effect of NME8 on reproductive toxicity under CP treatment. We will also analyze the expression of NME8 in the testes or the sperm of cancer patients before and after CP treatment or between CP treatment and untreated cancer patients. These may provide an experimental basis for improving the fertility of male patients after CP treatment.

## Materials and methods

### Animals

The animals were maintained under appropriate conditions (22 ± 1°C, 50–60% humidity, 12-hour light-dark cycle) throughout the study. The control group mice received an intraperitoneal injection of normal saline, while the CP group mice received an intraperitoneal injection of 5 mg/kg CP (Solarbio, Beijing, China) once a week for two or four weeks [19]. After the second or fourth administration for six days, the mice were euthanized by spinal dislocation, and their testes and epididymis were collected. No blinding was done for animal studies. The mice in this study were all randomized from male mice.

### Generation of *Nme8*^*−/−*^ mice

*Nme8*^*−/−*^ C57BL/6 mice were generated using clustered regularly interspaced short palindromic repeats (CRISPR)/Cas9 technology. Two guide RNAs (gRNAs) were designed to knock out the exon 6 - 7, the sgRNA target sequences were 5′-ACAGCACATTACCACCCTACAGG-3′ and 5′-ACACATAGAGAATCCAGTAAAGG-3′. Mutations near the sgRNA target sites were detected by PCR (primers were shown in Table S1) and gene sequencing analysis.

### Fertility test

The reproductive capacity of male mice was tested by co-caging them with female mice. One WT or *Nme8*^*−/−*^ adult male mouse (2–5 months old) was co-caged with two adult WT female mice for a period of three months. Three adult male mice were tested in each group, and the birth dates and litter sizes of the offspring were meticulously documented.

### Histological analysis

The testes and epididymis of the mice were placed in the appropriate volume of the Animal Testicular Tissue Fixative (Servicebio, Wuhan, China) to fix overnight at 4 °C. The fixed tissue samples were dehydrated in gradient ethanol and embedded in paraffin before being sectioned to a thickness of 5 μm. The paraffin sections were dried overnight. After deparaffinization and gradient rehydration, the paraffin tissue sections were stained with hematoxylin and eosin (H&E).

### Computer-assisted sperm analysis

The cauda epididymis of the mice was finely chopped in 400 μL M2 medium (Sigma-Aldrich, Missouri, USA) and then incubated at 37 °C for 30 min to release the sperm completely. Then, 10 μL of the fluid was placed on a sperm counting plate and examined using the Computer-Assisted Sperm Analysis (CASA) system (Tsinghua Tongfang Co., Ltd., Beijing, China) at 20× magnification. The sperm concentration, proportion of progressive sperms, nonprogressive motility, and immobilized sperms were recorded. At least 200 sperm cells were counted, and the process was repeated three times for each mouse.

### Immunol staining

Paraffin tissue sections were dewaxed in xylene and rehydrated in gradient alcohol, followed by antigen retrieval in 0.01% sodium citrate buffer (Solarbio, Beijing, China) for 20 minutes, and permeabilization with 0.2% Triton X-100 (Solarbio, Beijing, China) for 10 min. Subsequently, the sections were blocked with 5% goat serum at 37 °C for 30 min and then incubated with the primary antibody overnight at 4 °C. For immunofluorescence (IF) staining, primary antibodies were thoroughly washed off using PBS and incubated with fluorescent secondary antibodies (1:200; Invitrogen, California, USA) for 1 hour at 37 °C in the dark. After removing the secondary antibodies, the nuclei were stained with DAPI. Images were captured using an LSM900 confocal laser scanning microscope (Zeiss, Oberkochen, Germany). For immunohistochemical staining (IHC), after washing with PBS to remove the primary antibodies, the subsequent staining steps were completed using an immunohistochemical kit (Zsbio, Beijing, China) according to the manufacturer’s instructions. The antibodies used in this study included: DDX4 (1:200; ab27591, Abcam, Cambridge, UK), PCNA (1:50; sc-53407, Santa, Texas, USA), γH2AX (1:200; ab26350, Abcam, Cambridge, UK), c-KIT (1:200; AF1356, R&D, Minnesota, USA), NME8 (1:200, NBP3-15525, Novus, Colorado, USA), 4-HNE (1:200; ab46545, Abcam, Cambridge, UK), and SOX9 (1:200; A19710, ABclonal, Wuhan, China).

### Western blot

Total proteins from the mouse testes were extracted using RIPA lysis buffer (ThermoFisher Scientific, Massachusetts, USA) containing 1% PMSF. An appropriate volume of 5× loading buffer (Beyotime, Shanghai, China) was added to the total protein, and the mixture was boiled for 10 min. The protein samples were then separated by SDS-PAGE (10% or 12.5% polyacrylamide gels) and transferred to polyvinylidene difluoride membranes (Millipore, Massachusetts, USA). The membranes were blocked with 5% skim milk for 2 h at room temperature and then incubated with primary antibodies overnight at 4 °C. Following thorough washing to remove unbound primary antibodies, the membranes were incubated with secondary antibodies for 1 hour at room temperature. The image was developed using the Spark ECL super kit (Sparkjade, Shandong, China) and the Tanon 5200 Fully Automatic Chemiluminescence Image Analysis System. Protein bands were quantified using ImageJ software. The primary antibodies in the study included: NME8 (1:1000, NBP3-15525, Novus, Colorado, USA), 4-HNE (1:1000; ab46545, Abcam, Cambridge, UK), ALOX15 (1:1000; #DF13494, Affinity Biosciences, Jiangsu, China), GPX4 (1:1000; #DF6701, Affinity Biosciences, Jiangsu, China), γH2AX (1:1000; ab26350, Abcam, Cambridge, UK), LC3 (1:1000; 14600-1-AP, Proteintech, Wuhan, China), ATG5 (1:1000; ab108327, Abcam, Cambridge, UK), P62 (1:1000; #AF5384, Affinity Biosciences, Jiangsu, China), GAPDH (1:2000; 10494-1-AP, Proteintech, Wuhan, China). The full length uncropped original western blots used in this manuscript are available in the “Original Western Blot Images” file.

### Quantitative Real-time PCR

Total RNA was extracted from the testes using the Trizol reagent (Invitrogen, Carlsbad, USA), and reverse transcribed using a reverse transcription kit (Toyobo, Osaka, Japan). qRT-PCR was performed on a Roche fluorescence quantitative PCR instrument (LC480, Roche, USA). The 2 ^−ΔΔCt^ method was used to calculate the relative quantitative expression, normalized to the expression of Gapdh. The primers used in this study were shown in Table S2.

### Oxidative damage testing

Various assay kits were employed to identify different cellular redox markers. The experiments were carried out according to the provided instructions by the assay kits. The assay kits used were the Glutathione-s-transferase (GST) Activity Assay Kit (Mlbio, Shanghai, China), Thioredoxin peroxidase (TPX) Activity Assay Kit (Mlbio, Shanghai, China), Malondialdehyde (MDA) Assay Kit (Mlbio, Shanghai, China), and Total Superoxide Dismutase (SOD) Assay Kit with NBT (Beyotime, Shanghai, China).

### ROS levels

After sperm and testicular cells from each group of mice were collected, the 2′,7′-dichlorodihydrofluorescein diacetate (DCFH-DA) in the Reactive Oxygen Species (ROS) Assay Kit (Beyotime, Shanghai, China) was added. Incubated at 37 °C for 20 min and centrifuged at 2600 rpm for 5 min. Cells were isolated and resuspended with PBS at the same volume. A Microplate Reader (VICTOR3, PerkinElmer, Finland) was used to detect the fluorescence signal with an excitation wavelength of 488 nm.

### 8-hydroxy-2′-deoxyguanosine (8-OHdG) concentration

Testicular tissues were crushed in pre-cooled saline and centrifuged at 13,000 rpm for 30 min. In total 10 μL of the supernatant was then added to the reaction wells of the Mouse 8-OHdG ELISA Kit (Y-S Bio-Technology, Shanghai, China), followed by the addition of 40 μL of sample diluent. The reaction wells with samples were incubated at 37 °C for 1 h. The procedure continued according to the manufacturer’s instructions for plate washing, color development, reaction termination, and absorbance measurement at 450 nm using the Microplate Reader (VICTOR3, PerkinElmer, Finland). The concentration was then calculated according to the standard curve.

### TUNEL Assay

Apoptosis in testicular tissues was detected using the One Step TUNEL Apoptosis Assay Kit (Beyotime, Shanghai, China). Paraffin tissue sections were dewaxed in xylene, rehydrated in gradient alcohol, and incubated with protease K working solution for 30 minutes at 37 °C. After washing three times in PBS, the sections were incubated with TUNEL detection solution for one hour at 37 °C in the dark. The nuclei were stained with DAPI and sealed with an anti-fluorescence quenching agent. Images were captured using an LSM900 confocal laser scanning microscope (Zeiss, Oberkochen, Germany).

### Sperm chromatin dispersion (SCD) assay

The Sperm Chromatin Dispersion kit (GENMED, Surrey, UK) was employed to assess the level of sperm nuclear DNA fragmentation. The fluorescence microscopy was utilized to observe the halo size of the sperm head. A halo width exceeding 1/3 of the sperm nucleus diameter indicated the absence of DNA fragmentation. Conversely, a halo equal to or less than 1/3 of the sperm nucleus diameter indicated positive DNA fragmentation. Meanwhile, the absence of a halo or the presence of irregular or weak fluorescence in the nucleus center was indicative of severe DNA fragmentation. More than 200 sperms were counted per mouse.

### Statistical analysis

The data were presented as mean ± standard deviation (SD), and statistical analyses were conducted using the student’s t-tests, and *P* < 0.05 was considered statistically significant. More than three replicates were conducted to ensure reproducibility. The sample sizes are displayed in the figure legends. The graphs in this study were created by GraphPad Prism software. Experimental sample size was estimated based on our previous experience performing similar studies in mice. There was no data exclusion.

## Supplementary information

Supplemental material

Full-length uncropped original western blots

## Data Availability

The datasets generated and analyzed during the current study are available from the corresponding author on reasonable request.
