# Supplementary material for: Nme8 is essential for protection against chemotherapy drug cisplatin-induced male reproductive toxicity in mice
Source: Cell Death Dis. 2024 Oct 6;15(10):730. doi: 10.1038/s41419-024-07118-2 (PMC11457495; doi:10.1038/s41419-024-07118-2)
Supplement: Supplementary file 1 — Supplemental material [file 41419_2024_7118_MOESM1_ESM.docx]

**Table S1. The primers of Nme8 used for PCR in this study.**

| **Primer name** | **Sequence** |
| --- | --- |
| F1 | 5′-CAACCAGTTCTACGGGAGCA-3′ |
| R1 | 5′-GATGCCCCCATGTTTGTGAT-3′ |
| F2 | 5′-TGTACCGGAAAACGCCAGAA-3′ |
| R2 | 5′-ATCTTGCTCAGTGCCTGGTG-3′ |
| F3 | 5′-CAACCAGTTCTACGGGAGCA-3′ |
| R3 | 5′-AGCCAGGCAAGTTTTCATCTC-3′ |
| F4 | 5′-AGGGTGCAAATGCTCCACTTA-3′ |
| R4 | 5′-CGTCCTCGACATCGCAGTAA-3′ |
| F5 | 5′-CCTGACTTTGAAGAGTTTGTCG-3′ |
| R5 | 5′-TTGAGCTTCTGTGAATTCTTTCTT-3′ |

**Table S2. The primers used for qRT-PCR in this study.**

| **Gene** | **Forward primers** | **Reverse primers** |
| --- | --- | --- |
| Nme8 | 5′-CAACCAGTTCTACGGGAGCA-3′ | 5′-AGCCAGGCAAGTTTTCATCTC-3′ |
| Nme5 | 5′-TGTACCGGAAAACGCCAGAA-3′ | 5′-GGGGCTCAGGTGTAGTTTCC-3′ |
| Nme6 | 5′-ATCTTGCTCAGTGCCTGGTG-3′ | 5′-AGGTCATCTCTGCCCTGGAT-3′ |
| Nme9-NDPK | 5′-CCTTCCTGGGACCTTGTGAC-3′ | 5′-CTCTGTTGGCGTCCTCTCTG-3′ |
| Nme9-TRX | 5′-AGGACTGACTGTTGTGGACG-3′ | 5′-CCTCTGCCGAAGCAAAATGG-3′ |
| Gapdh | 5′-GATGCCCCCATGTTTGTGAT-3′ | 5′-GGCATGGACTGTGGTCATGAG-3′ |


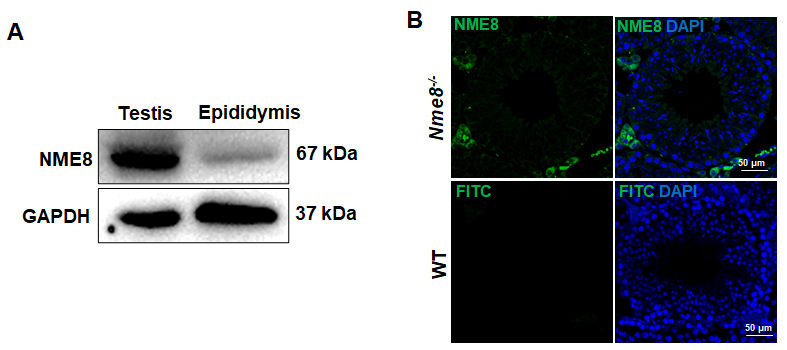


**Figure S1. Expression of NME8 in testis and epididymis.** (A) The protein level of NME8 in testis and epididymis of WT mice. (B) The negative controls for Figure 1K. The first row showed *Nme8^-/-^* mouse testes incubated normally with primary and secondary antibodies, and the second row showed WT mouse testes incubated with secondary antibodies only. Scale bar = 50 μm.


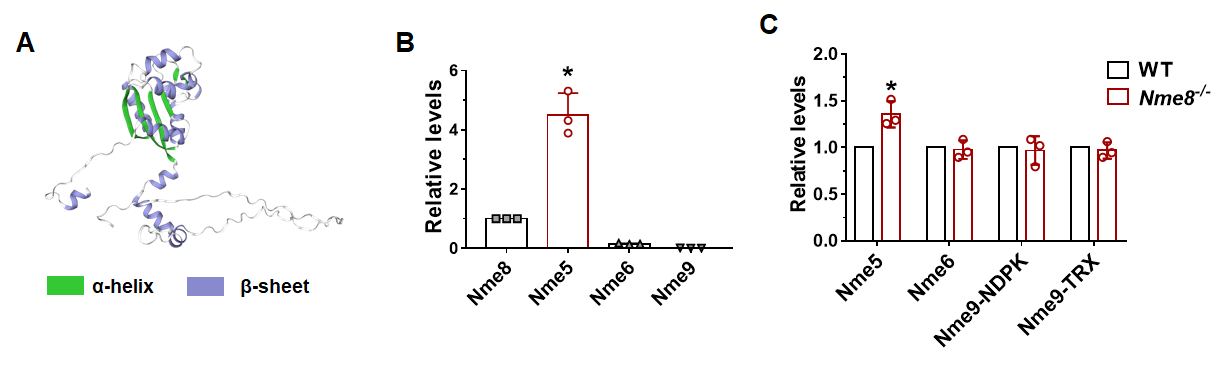


**Figure S2. Protein structure and related genes expression of *Nme8*.** (A) The predicted protein structure of NME8 in *Nme8^-/-^* mice. (B) The mRNA levels of *Nme8*, *Nme5*, *Nme6*, and *Nme9* in WT mice testis. (C) The mRNA expression levels of *Nme8* related genes in the testis of WT and *Nme8^-/-^* mice. **P* < 0 .05, n = 3 biologically independent mice in each group.
