# Supplementary figures and images for: Nme8 is essential for protection against chemotherapy drug cisplatin-induced male reproductive toxicity in mice
Source: Cell Death Dis. 2024 Oct 6;15(10):730. doi: 10.1038/s41419-024-07118-2 (PMC11457495; doi:10.1038/s41419-024-07118-2)

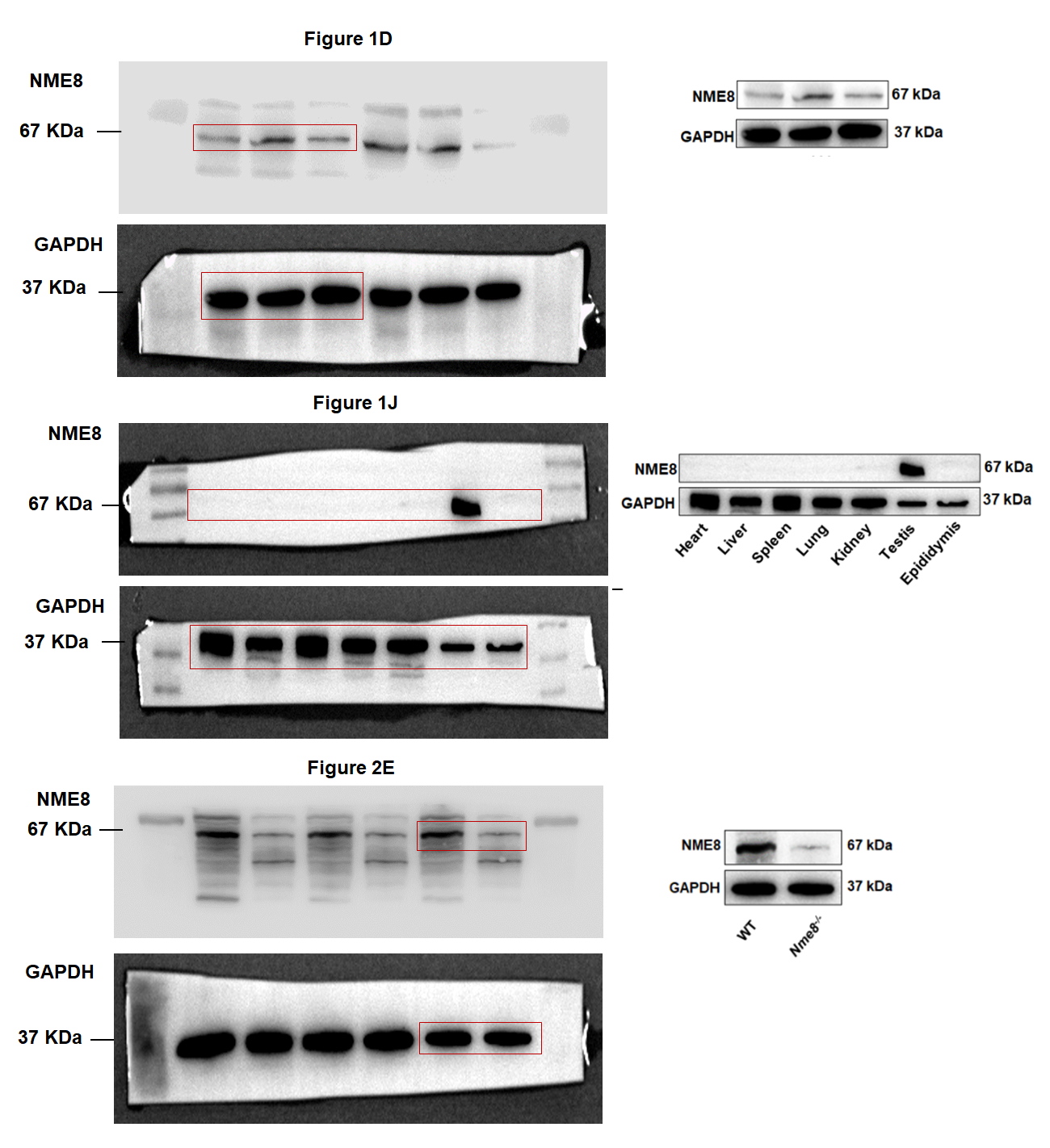


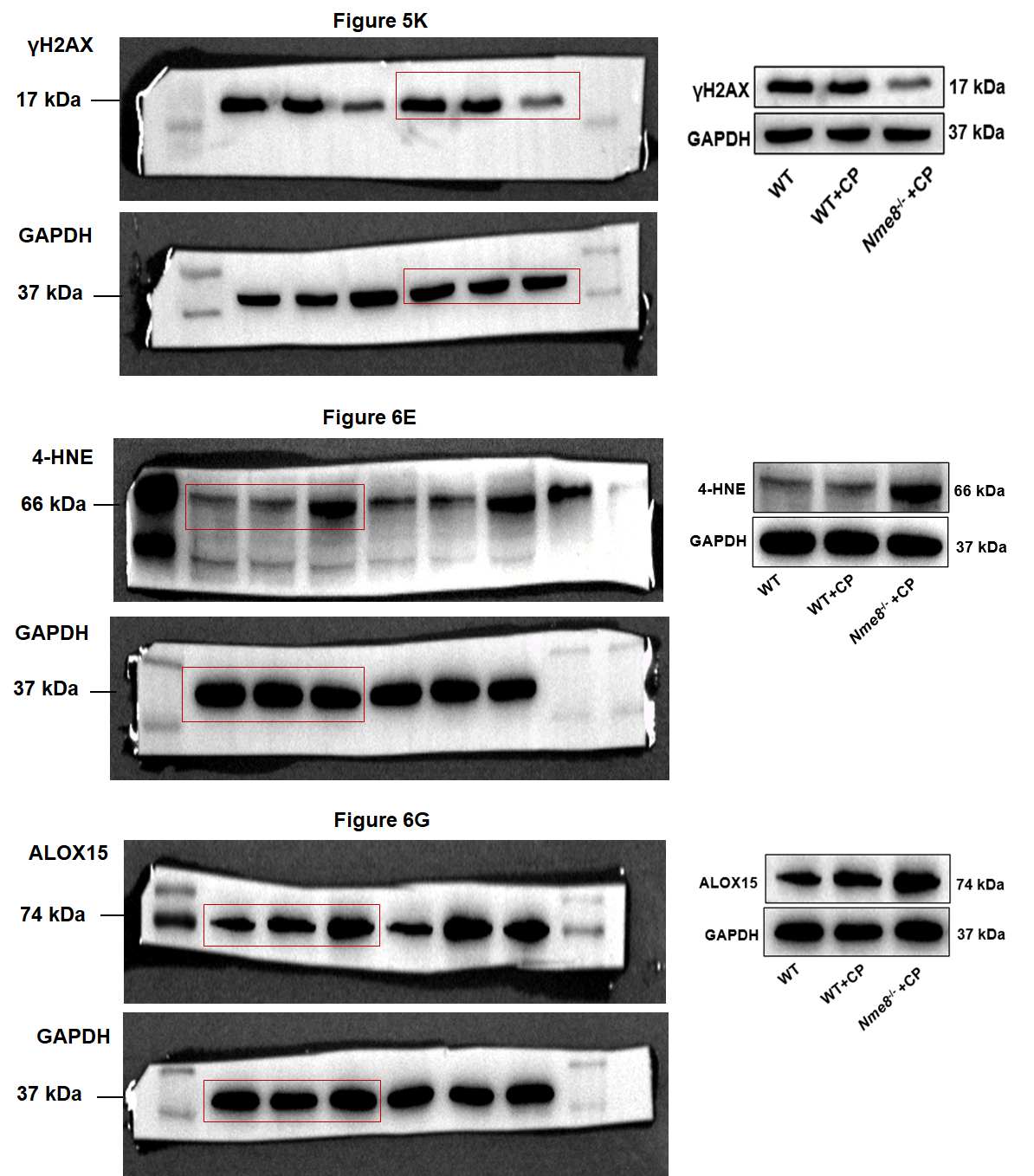

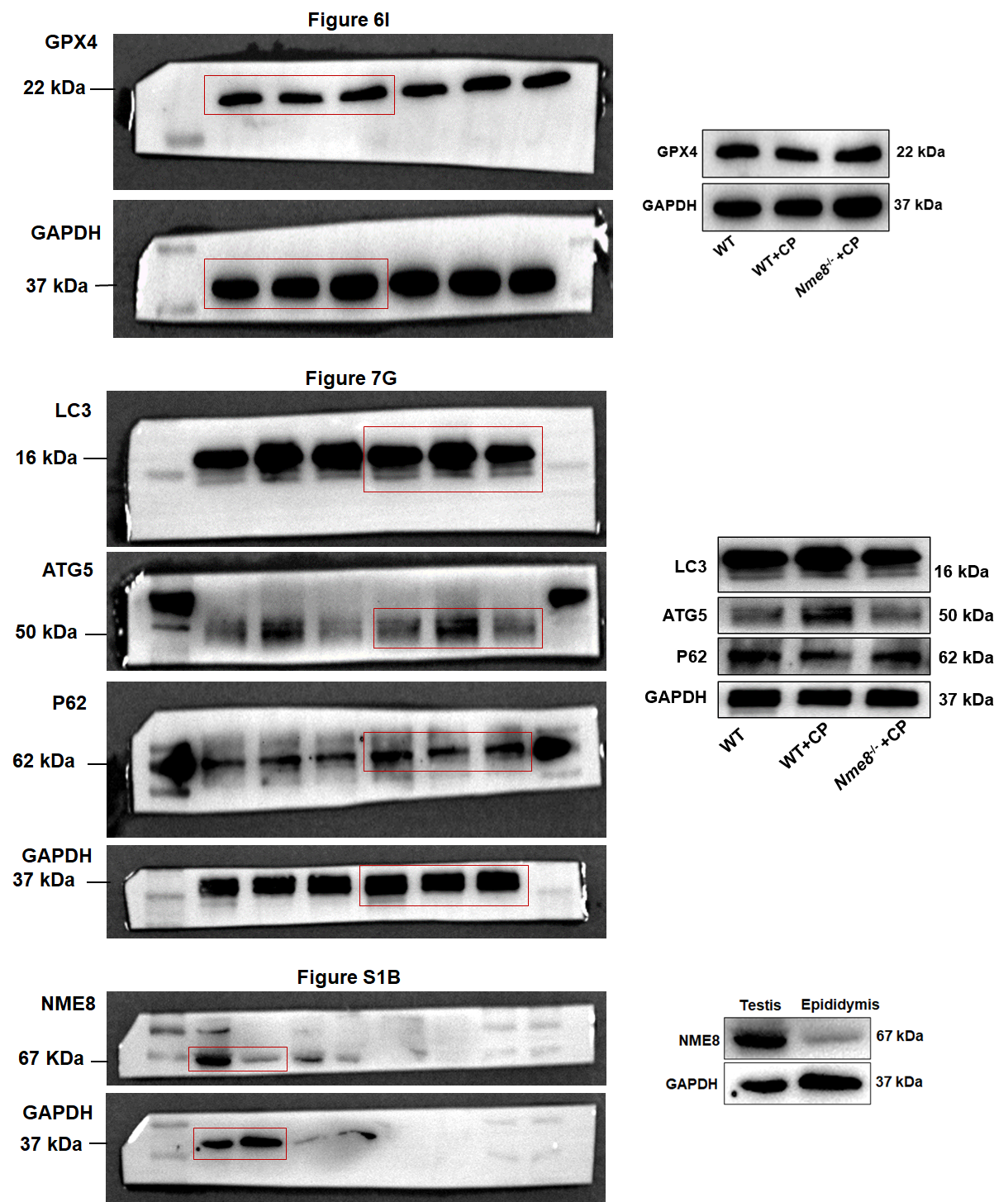

Supplement: Supplementary file 2 — Full-length uncropped original western blots [file 41419_2024_7118_MOESM2_ESM.docx]
